# The effect of ambient temperature on blood pressure in a rural West African adult population: a cross-sectional study

**Published:** 2010-02

**Authors:** Setor K Kunutsor, John W Powles

**Affiliations:** Malaria Consortium, Comdis Muk, Kampala, Uganda; Institute of Public Health, University of Cambridge, Robinson Way, Cambridge, UK

**Keywords:** blood pressure, ambient temperature, rural, seasons

## Abstract

**Introduction:**

Associations between ambient temperature and blood pressure have been demonstrated in countries where the temperature varies between the seasons. This phenomenon has been overlooked in blood pressure surveys in sub-Saharan Africa. We assessed the effect of ambient temperature on blood pressure in an adult population in a West African country.

**Methods:**

A cross-sectional survey was carried out on a rural Ghanaian population, investigating the effect of ambient temperature on blood pressure in 574 randomly sampled adults aged between 18 and 65 years.

**Results:**

There was a significant inverse relationship between ambient temperature and systolic (SBP) (*p* < 0.019) and diastolic blood pressure (DBP) (*p* < 0.036). SBP fell by 5 mmHg per 10ºC rise in ambient temperature.

**Conclusion:**

Higher ambient temperatures are associated with lower blood pressures. To enhance comparability of data from epidemiological surveys, ambient temperature should be recorded for each blood pressure reading and findings standardised to a fixed ambient temperature.

## Summary

Seasonal variations in blood pressure have been observed among adults, children and the elderly in different countries, suggesting that blood pressure is influenced by temperature changes. Blood pressures have been found to be higher in winter and lower in summer.^1^-^4^ Woodhouse *et al.*^5^ in their study of an elderly population to determine the seasonal variation in blood pressure and its relationship to ambient temperature reported that both systolic (SBP) and diastolic blood pressures (DBP) were highest during the winter across the whole distribution of blood pressure readings. Hopman and Remen^6^ demonstrated in 1921 that BP was higher in the colder than the warmer months. Kauffmann^7^ showed in 1924 that SBP was 20 to 30 mmHg higher on cool days than at other times. Brown^8^ demonstrated the lowering of BP in men during the summer period, which was also demonstrated by Rose.^9^ A study conducted among children in Australia showed that a change of 10ºC in temperature caused an estimated difference in BP of 5 to 7 mmHg.^10^

Despite the extent of evidence on the ambient temperature– blood pressure phenomenon, not much attention has been paid to its implications for clinical practice, and quantitative formulations have not been developed to correct for this bias. There are currently no data published on the relationship between ambient temperature and blood pressure in sub-Saharan West African countries, where the seasons are classified into two – the rainy and dry seasons. Daily maximum temperatures during the dry season range between 38 and 45ºC and those for the rainy season are between 20 and 26ºC.

The aim of this study, part of a cross-sectional blood pressure survey, was to study the influence of ambient temperature on blood pressure in a rural West African adult population.

## Methods

The study was conducted in the Kassena–Nankana District (KND) of northern Ghana. Ghana is a tropical country located on West Africa’s Gulf of Guinea, a few degrees north of the Equator, with a total area of 238 540 km2. It has an estimated population of 19.7 million, a gross domestic product (GDP) of 7.4 billion US dollars and a per capita GDP of 412 US dollars.

KND is one of the eight districts in the northernmost part of Ghana and Navrongo is its commercial and political capital. It is a relatively flat country with an area of 1 675 km2, which is wholly in the sub-sahelian woodland of West Africa. The vegetation is guinea savannah, which is characterised by short scattered trees and a more or less continuous carpet of grasses. The mildly cool rainy season extends from April to September and the prolonged and intensely hot, dry season is from October to March. Rainfall averages from 850 to 950 mm per year, with the peak occurring in August. Daily mean temperatures range between an average minimum of 20ºC in the rainy season and an average maximum of 40ºC in the dry season. In the dry season, early mornings are usually cool and the afternoons commonly hot with daily maximum temperatures going as high as 45ºC.

This was part of a cross-sectional survey to determine the blood pressure levels in a rural West African population. A random sample of 600 participants was drawn from a population register, regularly updated by the Navrongo Demographic Surveillance System (NDSS), which is owned by the Navrongo Health Research Centre, a field station of the Health Research Unit of the Ghana Health Service. NDSS is a continuous population registration system that assesses the demographic dynamics of the entire population of the Kassena–Nankana district. Every 90 days, all compounds in the district are visited to update vital events such as births, deaths, pregnancy, marriages and migration in and out of the district.

Blood pressure, anthropometric, time of blood pressure and room temperature measurements were taken in 574 adult males and females aged between 18 and 65 years, resident in the study area and who agreed to be part of the study. Response rate was 95.7% with 207 males and 367 females completing the study protocol. The imbalance between the genders in the random sample drawn was due to the fact that females constitute a larger percentage of the population. The 26 non-respondents, all males, were either busy on their farms or not interested in participating. Fieldwork was carried out in the KND from February to April 2007, the period when morning temperatures are low and daily temperatures reach their maximum.

Ethical approval was obtained from the local Institutional Review Board of the Navrongo Health Research Centre, Navrongo, Ghana (reference: NHRC 061). The traditional chiefs, community leaders, political and opinion leaders were approached prior to commencement of fieldwork, and community meetings were held to explain the purpose of the whole study. Individual affirmations of informed consent were obtained from residents willing to participate, using informed consent forms. The consent forms were signed by those who were literate and marked with a left thumbprint for those who were not.

Five fieldworkers and a supervisor with prior experience and training in epidemiological surveys of this nature were recruited and retrained in the techniques of questionnaire administration, and anthropometric and blood pressure measurements. The study team were all locals of the study area and were fluent in the local languages. Before the start of the fieldwork, each of the techniques and instruments was tested on a small sample of the population and shown to be practicable, sufficiently reliable and valid.

Data were collected by interview using a simple structured questionnaire, and measurements were recorded on a standard form. For date of birth, an estimated date was assigned to those who were unable to give their exact dates of birth, using the seasons, festivals and some historical indicators as guides. June 30 was allocated if the exact date and month could not be decided upon.

Blood pressure measurements followed administration of the questionnaire and anthropometric measurements. Reliable automatic blood pressure devices (Omron MX3 plus), which were validated against the reference method of sphygmomanometry (have passed the validation recommendations of the International Protocol of the European Society of Hypertension),^11^ were used by trained staff. Measurement was taken in the quiet room provided, and the room temperature and time of the day was recorded. Blood pressure was measured in the right arm with participants in the seated position. Blood pressure and temperature measurements were taken between 07:00 and 14:00 for the main survey.

Two weeks after the main survey, a sub-sample, randomly sampled from the study participants, were invited to have repeat BP and temperature measurements taken in the morning between 06:00 and 10:00 when temperatures were at their minimum, and again in the afternoon between 13:00 and 15:00 when the temperatures were at their maximum. Thirty-seven participants volunteered for the repeat measurements. In the repeaters, BPs and time-of-day measurements were taken randomly. Following the WHO MONICA methodology,^12^ blood pressure was measured twice and the mean of the two used for the analysis. The same trained staff took blood pressure measurements in all locations.

## Statistical analysis

To determine BP changes with ambient temperature, readings were grouped based on the temperatures at the time of blood pressure measurements in the morning, and in the afternoon for the follow-up survey. Two temperature categories were used. Category 1 consisted of BP readings with an ambient temperature range of 28 to 34ºC and category 2 consisted of readings with temperatures ranging from 39 to 43ºC. BP readings for the two temperature groups were averaged and compared.

Pearson’s linear correlation was performed to determine the dose–response relationship between ambient temperature and BP readings. To further explore the association between temperature and BP, bivariate regression analyses were used. To assess the independent association of ambient temperature with BP variability, systolic and diastolic blood pressure were regressed on ambient temperature in separate multiple linear regression models, including and excluding age, gender, body mass index, waist circumference and time of blood pressure measurement. *P*-values < 0.05 (two-sided) were considered statistically significant. All results were given as mean (standard deviation) unless indicated otherwise. All statistical analyses were performed using the Statistical Package for Social Sciences (SPSS) version 15 for MS WINDOWS.

## Results

The 574 participants ranged in age from 18 to 65 years. Mean age was 37.8 (14.0) years with 36.1% being males and 63.9% females. Mean age of males was 36 (14) years and that of females was 39 (14) years. The sub-sample of 37 participants ranged in age from 18 to 65 years. Mean age was 38.4 (14.8) years.

For the follow-up survey, at morning temperatures of between 28 and 34ºC, mean SBP and DBP were 123.1 (18.4) and 71.9 (13.0) mmHg, respectively. At afternoon temperatures between 39 and 43ºC, mean SBP and DBP were 120.9 (19.4) and 70.6 (12.1) mmHg, respectively. There was a moderate but relevant difference in mean systolic and diastolic BPs for the two ambient temperature categories.

**Fig. 1.**
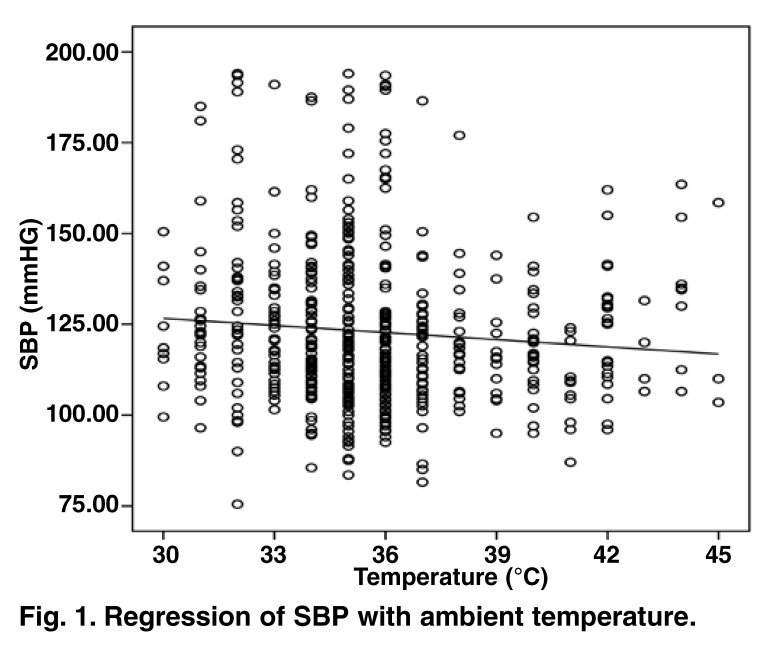
Regression of SBP with ambient temperature.

Mean correlation coefficients for SBP and DBP against temperature are displayed in (Table 1). A significant inverse dose–response relationship was demonstrated between ambient temperature and BP (for both SBP and DBP). The significant inverse correlation between SBP and ambient temperature is illustrated graphically in Fig. 1 (*r* = –0.1, *p* = 0.02).

**Table 1:** Correlation Of Blood Pressure With Ambient Temperature

*Variable*	*Pearson’s correlation (r)*	p*-value*
SBP (mmHg)	–0.098*	0.019
DBP (mmHg)	–0.088*	0.036

*Correlation significant at *p* = 0.05.

Linear regression analysis showed that SBP was significantly and inversely related to ambient temperature (β = –0.98, *p* = 0.02, 95% confidence interval: –1.19 to –0.11). Table 2 shows results of the multiple regression model which included ambient temperature. The results show that for the given variables in the model, SBP was lowered by 5 mmHg per 10ºC increase in temperature. The results are in favour of significant changes in blood pressure when exposed to different ambient temperatures.

**Table 2:** Multiple Regression Analysis Of SBP With Age, Waist Circumference, Alcohol Consumption, Smoking And Temperature

*Model*	*β*	*SE*	*p-value*
(Constant)	100.812	13.096	0.000
Age (years)	0.332	0.062	0.000
Waist circumference (cm)	0.411	0.090	0.000
Alcohol (yes or no)	–3.003	1.758	0.088
Smoking (yes or no)	–0.362	2.832	0.898
Temperature (ºC)	–0.521	0.262	0.047

*r*^²^ = 0.119; β: regression coefficient; SE: standard error; dependent variable: SBP.

## Discussion

The results presented in this study show a significant influence of ambient temperature on blood pressure measurements. An inverse dose–response relationship was demonstrated for both SBP and DBP with ambient temperature, but this was more significant for SBP. Jansen *et al.*^13^ in their study of the effect of change in ambient temperature on BP demonstrated an inverse dose–response relationship between BP and temperature but more especially for SBP. The regression analysis also showed significant changes in SBP with ambient temperature.

Other factors that could have caused the variation in BP during specific periods of the day should be considered when interpreting the results. A difference in stress levels could have been a possible confounder but this could not have been so for this rural population because their stress levels are usually high in the daytime when they go to the farm and it is expected that their blood pressures would have been high around this time. Daily variation in BP is also a potential confounder but this is unlikely, considering the significant variation in BP with temperature. Other potential confounding sources include lifestyle factors such as diet, but these are highly unlikely considering the cross-sectional nature of the study. Ambient temperature appears to be the main factor causing the variation in BP.

The seasonal variation in SBP and DBP has been documented in many studies,^5^-^10^ with ambient temperature being implicated in this variation.^14^,^15^ Jansen and colleagues^13^ demonstrated a moderate but significant influence of ambient temperature on BP. A significant increase in both SBP and DBP was seen when moving from higher to lower ambient temperature. Komulainen and colleagues^16^ in their study also reported that BP seems to react in three minutes to changes in ambient temperature. Chifamba *et al.*^17^ studied the effect of variation in environmental temperature on blood pressure in subjects in Zimbabwe and showed that SBPs and DBPs were significantly higher when recorded at 15ºC than at 25ºC (a mean difference of 32.2 ± 4.2 mmHg and 19.5 ± 3.0 mmHg for SBPs and DBPs, respectively).

It has been observed that people with high blood pressure require less medication in the summer compared with the winter.^18^ Various studies have also reported increased prevalence of strokes and myocardial infarctions and higher cardiovascular disease mortality during the winter months.^19^,^20^ The phenomenon of BP variation with ambient temperature demonstrated in this study is a possible explanation for these observations.

Several mechanisms have been suggested to explain this phenomenon. One is the activation of the sympathetic nervous system. Cold temperature increases sympathetic tone and increased secretion of catecholamines, which in turn causes increased heart rate and BP. The BPs associated with warm temperature is due to cutaneous vasodilatation, which in turn reduces the peripheral vascular resistance and BP.

## Study limitations

Our study was subject to multiple potential biases. The use of the automated blood pressure device (Omron MX3 Plus) opposed to the random-zero sphygmomanometer recommended by the WHO MONICA protocol^12^ may have introduced measurement biases. However, the Omron MX3 Plus has been validated against the reference method of sphygmomanometry and has passed the validation recommendations of the International Protocol of the European Society of Hypertension, making it eligible for use in epidemiological surveys.^11^ Validated automated electronic BP devices may actually improve reliability and reproducibility of BP measurements.^21^ The devices were also regularly standardised against mercury sphygmomanometers to check for any drifts. Ideally 24-hour ambulatory, clinic or home blood pressure monitoring would have been the best way to demonstrate ambient temperature variations in blood pressure.

## Conclusion

Awareness of the phenomenon that there is a correlation between BP and ambient temperature is quite important, considering the fact that high blood pressures are recorded during low ambient temperatures. The significant differences in blood pressure at different ambient temperatures may have some implications, such as under- or overestimating blood pressure levels in various populations.
